# Glutathione Depletion and Carbon Ion Radiation Potentiate Clustered DNA Lesions, Cell Death and Prevent Chromosomal Changes in Cancer Cells Progeny

**DOI:** 10.1371/journal.pone.0044367

**Published:** 2012-11-20

**Authors:** Maïté Hanot, Anthony Boivin, Céline Malésys, Michaël Beuve, Anthony Colliaux, Nicolas Foray, Thierry Douki, Dominique Ardail, Claire Rodriguez-Lafrasse

**Affiliations:** 1 Laboratoire de Radiobiologie Cellulaire et Moléculaire, EMR3738, Faculté de Médecine Lyon-Sud, Oullins, France; 2 Fondation Synergie Lyon Cancer, Lyon, France; 3 Institut de Physique Nucléaire de Lyon, UMR 5822, Université Lyon 1, IN2P3/CNRS, Villeurbanne, France; 4 Institut National de la Santé et de la Recherche Médicale, U836, Groupe de Radiobiologie, Faculté de Médecine de Lyon-Sud, Oullins, France; 5 Commissariat à l'Energie Atomique (CEA), Service de Chimie Inorganique et Biologique UMR-E 3 (CEA-UJF), Laboratoire Lésions des Acides Nucléiques, Grenoble, France; 6 Unité Médicale d'Oncologie Moléculaire et Transfert, Hospices Civils Lyon, Centre de Biologie Sud, Centre Hospitalier Lyon-Sud, Pierre Bénite, France; University of Medicine and Dentistry of New Jersey, United States of America

## Abstract

Poor local control and tumor escape are of major concern in head-and-neck cancers treated by conventional radiotherapy or hadrontherapy. Reduced glutathione (GSH) is suspected of playing an important role in mechanisms leading to radioresistance, and its depletion should enable oxidative stress insult, thereby modifying the nature of DNA lesions and the subsequent chromosomal changes that potentially lead to tumor escape.

This study aimed to highlight the impact of a GSH-depletion strategy (dimethylfumarate, and l-buthionine sulfoximine association) combined with carbon ion or X-ray irradiation on types of DNA lesions (sparse or clustered) and the subsequent transmission of chromosomal changes to the progeny in a radioresistant cell line (SQ20B) expressing a high endogenous GSH content. Results are compared with those of a radiosensitive cell line (SCC61) displaying a low endogenous GSH level.

DNA damage measurements (γH2AX/comet assay) demonstrated that a transient GSH depletion in resistant SQ20B cells potentiated the effects of irradiation by initially increasing sparse DNA breaks and oxidative lesions after X-ray irradiation, while carbon ion irradiation enhanced the complexity of clustered oxidative damage. Moreover, residual DNA double-strand breaks were measured whatever the radiation qualities. The nature of the initial DNA lesions and amount of residual DNA damage were similar to those observed in sensitive SCC61 cells after both types of irradiation. Misrepaired or unrepaired lesions may lead to chromosomal changes, estimated in cell progeny by the cytome assay. Both types of irradiation induced aberrations in nondepleted resistant SQ20B and sensitive SCC61 cells. The GSH-depletion strategy prevented the transmission of aberrations (complex rearrangements and chromosome break or loss) in radioresistant SQ20B only when associated with carbon ion irradiation. A GSH-depleting strategy combined with hadrontherapy may thus have considerable advantage in the care of patients, by minimizing genomic instability and improving the local control.

## Introduction

Carbon ion hadrontherapy is highly effective for treating cancer located near critical organs at risk that is resistant to conventional radiotherapy, such as head-and-neck squamous cell carcinoma (HNSCC), because a more precise and powerful dose can be applied, leading to a high relative biological efficiency [1]. Carbon ions induce detrimental clustered damage comprising a combination of DNA double- and single-strand breaks (DSB and SSB), and abasic sites in the close vicinity of oxidized bases. In contrast to these carbon-ion-induced clustered lesions, X-rays induce rather sparse damage [2]. In both cases, misrepaired or unrepaired lesions may lead to chromosomal aberrations [3]–[5]. Some chromosomal changes transmitted to cell progeny may thus cause cancer cell adaptation [6] and tumor escape, the leading cause of radiotherapeutic failure. The growing interest in hadrontherapy for treating highly resistant cancers requires clarifying the impact of complex DNA lesions on the higher incidence of chromosomal changes (CCs). Identifying these processes would therefore be a major advance in the understanding of cancer recurrence, a well-known feature of radioresistant HNSCC [7]–[10].

DNA lesions and CCs are influenced by endogenous factors such as reactive oxygen species scavenging systems. A high level of endogenous reduced glutathione (GSH) often promotes cancer cell survival and resistance [11], and its depletion, investigated for decades along with radiotherapy, is cited today for new therapeutic considerations particularly for the treatment of cancers resistant to conventional or carbon ion radiotherapy [12]–[15]. Among other strategies, a GSH-depletion strategy may be used as a tool to modulate the nature, the number or the repair of DNA damage through oxidatively generated complex DNA damage [16]. Nevertheless, only limited and conflicting data are available regarding the relationship between GSH level and high linear energy transfer (LET) and low-LET radiation-induced DNA damage. For example, Mansour et al. [17] reported that *N*-acetylcysteine, a GSH precursor, protects hepatic tissues from radiation-induced DNA lesions, whereas other studies suggested a weak protector role of exogenous GSH on DNA lesions in lymphocytes or CHO cells [18], [19].

Studies of cytogenetic effects of high-LET radiation are helpful for analyzing the mechanisms underlying tumor cell radioresistance because they reflect the specificity, capacity and fidelity of repair or misrepair processes taking place in irradiated cells. The clustered DNA lesions induced by carbon ion irradiation are known to lead to highly complex chromosomal aberrations at metaphase [3], [5], but little is known about the risk of their transmission to cancer cell progeny, which is a potential cause of tumor escape. Although a relationship linking oxidative stress and genomic instability has been reported [20], [21], data related to the modulation of antioxidant defenses (such as GSH) are still conflicting. For example, whatever the radiation quality, an increase in the endogenous GSH pool may inhibit sister chromatid exchange [22], increase exchange aberrations [19], or reduce the frequency of aberrant metaphases [18]. Deletion is the only common type of CC reported in previous studies that demonstrate that the number of deletions correlates inversely with GSH level [18], [19], [23], [24]. Although an increasing number of data are available, with explanations or hypotheses linking these observations to the initial radioinduced DNA lesions, data on the repair capacity or the risk of CC transmission to the cell progeny are still lacking. Only Pujari et al. [19] have suggested that high-LET radiation combined with a GSH supplementation marginally influenced the chromosomal exchange frequency compared with X-rays, indicating that GSH failed to protect cells from DNA damage under these experimental conditions.

Cytogenetic studies have often led to conflicting data, probably because of the diversity of methods used, which estimated either early effects (during metaphase or with premature chromosome condensation techniques) [4], [18], [25] or late effects (on the progeny) [26], [27]. Measurement of the early effects raises the problem of the cell cycle shift caused by irradiation, leading to an underestimation of CCs when data are collected at only one time point and to a lack of indication concerning the effects on transmissible events [3], [4]. Moreover, cancer cells display a highly modified genome that limits observations and measurements of chromosomal aberrations using FISH-painting techniques [4]. Measurement of the late effects provides information on the transformation or differentiation in the progeny, but excludes events arising at the time of first mitosis [26], [27]. Thus, there has been, up to now, no clear consensus about which is the more relevant method for studying the risk of chromosomal aberration transmission in surviving cells.

An alternative method may provide a link regarding chromosomal instability between these different data and refers to the cytokinesis-block micronucleus assay that evolves into a “cytome assay” [28]–[31]. This assay is based on the morphological observations of nuclei after cytokinesis blockage, i.e. cells that have completed one nuclear division are identified by their binucleated appearance. It provides information on some CCs transmitted to daughter cells [32], [33] through micronucleus and nucleoplasmic bridge formation. This well-described assay is now considered as a reference test for monitoring human CCs and enables the identification of cells passing through the first delayed mitosis. Such transmissible aberrations may induce genomic instability in surviving cells, leading to tumor escape.

The aim of the present study was first to determine the quality and the quantity of DNA lesions in relation to endogenous GSH level and radiation quality, and second to determine the consecutive CCs in surviving cancer cells after X-ray and carbon ion irradiation in order to evaluate the risk of radioinduced instability and then tumor escape. A resistant HNSCC cell line (SQ20B) displaying a high endogenous GSH (∼70 nmol/mg protein) level was chosen as a study model. A transient GSH-depletion strategy was applied before irradiation, based on the use of dimethylfumarate (DMF), a glutathione-depleting agent, and buthionine sulfoximine (BSO), a glutathione biosynthesis inhibitor. This strategy has been previously tested [14] in terms of in vitro toxicity and the activated pathways leading SQ20B cells to death after irradiation were clearly demonstrated. It allowed SQ20B cells to display the same sensitivity as SCC61 cells, a radiosensitive HNSCC cell line that displays a low endogenous GSH content [14], [34]. In this paper, the data obtained from radioresistant SQ20B cells and GSH-depleted SQ20B cells were therefore compared with sensitive SCC61 cells. To compare events leading to an equivalent level of cell death, X-ray and 75 MeV/n carbon ion irradiation were used at biologically equivalent doses, assuming a relative biological efficiency of about 2 at 10% survival for both cell lines [34].

Taken together, our results lead to a new understanding of the quality and the number of DNA lesions in relation to GSH level and radiation quality and thus clarify some divergent results reported in the literature. In this regard, the cytome assay gave a clear overview of chromosomal aberrations transmitted in the surviving cancer cells. It enabled the assertion that an increase of the DNA lesion complexity obtained by GSH-depletion adjuvant therapy combined with hadrontherapy may minimize genomic instability in resistant cancer cells and thus reduce the phenomenon of tumor escape after radiotherapy.

## Materials and Methods

### Cell Culture and Treatments

SCC61 (SF2 = 0.36) and SQ20B (SF = 0.72) cell lines were cultured as described previously [34]. Cells were cultured for no more than 12 passages. Dimethylfumarate (DMF, 100 µM), a GSH-depleting agent, and l-buthionine sulfoximine (BSO, 100 µM), an inhibitor of GSH biosynthesis, were added to the SQ20B culture medium 4 h before irradiation to deplete GSH, as described previously [14]. In some experiments, *N*-acetyl cysteine (NaC, 5 µM) was also added to the culture medium 4 h before irradiation and in combination with the DMF/BSO treatment.

### Chemicals

Primary γH2AX and centromeric protein A (CENPA) mouse antibodies were obtained from Upstate and Abcam, respectively, and the secondary antibody AlexaFluor 488 goat anti-mouse IgG was obtained from Invitrogen. Antifade mounting medium was purchased from Dako. Low-melting point agarose and SYBR Green solution were from Sigma, and the formamidopyrimidine glycosylase (Fpg) was obtained from Trevigen.

### Irradiation Procedures

Monolayers of cultured cells were irradiated as described previously [35]: X-ray irradiation with 6 MV was performed in Lyon-Sud Hospital (Radiotherapy Department), France, on a Clinac CD irradiator at a dose rate of 2 Gy/min. Irradiation with 72 MeV/u carbon ions (LET 33.6 keV/μ) was performed at GANIL, Caen, France.

### Analysis of Clonogenic Cell Survival

Clonogenic cell survival was monitored after X-ray and carbon ion exposure at doses ranging from 1 to 5 Gy. The cells were seeded before irradiation and reseeded immediately after exposure into flasks of 25 cm^2^ at different concentrations. Cell survival was assessed by the standard colony formation assay as described in [35].

### HPLC Analysis

Total glutathione was quantified by HPLC analysis. Briefly, proteins were precipitated from the cellular homogenate with sulfosalicylic acid and centrifuged at 13.000×*g*. The supernatant was then derivatized with *o*-phthalaldehyde. Chromatographic separation was achieved on a 5 µm Spherisorb C18-column, with a mobile phase composed of methanol–0.15 M acetate buffer pH 7 (7.5∶92.5). Fluorescence of the glutathione-*o*-phthalaldehyde derivatives was detected at an emission wavelength of 420 nm and an excitation at 340 nm [36].

### Immunofluorescence

The detection of γH2AX foci or CENPA was assayed by immunohistochemistry. Briefly, cells were fixed in 3% paraformaldehyde for 20 min, and immunodetection was performed as described [37]. The CENPA detection was performed with the cytome assay described below. Digital images were obtained using a fluorescent microscope (Axio Imager Z1 Zeiss microscope, 400× magnification). A minimum of 100 nuclei were scored at each time to calculate the average number of γH2AX foci using ImageJ software.

### Single DNA Lesion Detection using Alkaline Single-Cell Gel Electrophoresis (SCGE)

Cells were trypsinized, centrifuged at 1000 rpm at 4°C, and the pellet was suspended in freezing medium (10% DMSO, 40% DMEM, 50% SVF) and stored at −80°C. After defrosting, the samples were centrifuged (1000 rpm, 4°C), and the pellet was suspended in cold PBS and mixed with 0.6% low-melting point agarose. Gels were spread onto microscope slides and the SCGE technique was used as described by Tice et al. [38]. Slides intended for oxidized DNA base measurements were washed with the enzyme buffer (0.1 M KCl, 10 mM EDTA, 10 mM HEPES-KOH, 0.02 mg/ml BSA, pH 7.4) and incubated for 20 min (37°C) with Fpg in the enzyme buffer or with buffer alone. All slides were then incubated for 40 min in an alkaline electrophoresis buffer (pH>13) to induce DNA unwinding. Electrophoresis was performed in the same buffer for 35 min at 25 V/300 mA at 4°C. The slides were rinsed with 400 mM Tris base (pH 7.5) to neutralize the excess alkali. After staining with the SYBR Green solution, nuclei “comets” were viewed on the Zeiss microscope. A total of 100 comets were observed visually and scored on each slide using free CASP software. The tail intensity, defined as the percentage of DNA migrating from the head of the comet into the tail, was measured for each scored nucleus.

### Cell Cycle Analysis

Propidium iodide (PI) staining was used to analyze the cell cycle distribution, as previously described [34]. Briefly, cells were fixed with 70% ethanol, incubated with 5 µg/ml PI (Sigma-Aldrich) and 0.5 mg/ml RNase A (Sigma-Aldrich), and then analyzed using a FACScan flow cytometer.

### Cytome Assay: Micronuclei (MN) and Chromosomal Rearrangements

The multiendpoint cytokinesis-blocked micronucleus assay was used to assess chromosome aberrations [32]. Cytochalasin B (5 µg/ml) was added to the culture medium in order to block cytokinesis and collect cells that had completed the first nuclear division. No binucleated cells are numbered in the 5 h following addition of cytochalasin B. The concentration selected as indicated above showed the highest frequencies of binucleated cells in controls (95%, 28 h after addition of cytochalasin B) and had no influence on the level of spontaneously occurring MN or nucleoplasmic bridges. Cytochalasin B was added 4 h before irradiation in order to cumulate the most representative cell population at a binucleated stage 24 h later. Cells were fixed in 3% paraformaldehyde and stained with DAPI (5 µg/ml). In these experiments, different endpoints were considered: binucleated cells with MN, centromere-positive MN, nucleoplasmic bridges (NPB), and cells with simultaneous NPB and MN (NPB+MN) [28], [39], [30]. These markers are described here in ascending order of complexity.

Cells with MN. These cells are characterized by the presence of both a main nucleus and one or more smaller nuclei called MN. The frequency of MN in the cell population is the yield of MN (Ymn), which is calculated as:
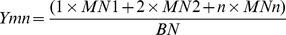
where MN1 is the number of cells with one micronucleus, MN2 the number of cells with two MN, MNn the number of cells with n MN, and BN is the total number of binucleated cells. The presence of MN is indicative of the loss of chromosome fragments that corresponds to acentric chromosome/chromatid resulting from unrepaired DNA breakage events. By contrast, MN containing one or more centromeres (immunodetected as CENPA) indicates chromatid/chromosome loss. The ratio of centromere-positive micronuclei (c+MN) was estimated as the percentage of binucleated cells with c+MN.

Cells with NPB. NPB are continuous DNA-containing structures linking the nuclei in a binucleated cell. NPB originate from dicentric chromosomes in which the centromeres are pulled to opposite poles during anaphase and are therefore representative of misrepaired DNA, chromosome rearrangement or telomere end fusion.

Cells with simultaneous expression of NPB and MN. The expression of NPB+MN in divided cells arises from break–fusion–break cycles and represents a highly complex rearrangement.

All scoring criteria were used according to the morphological parameters described by Fenech [33].

### Statistical Analysis

The data from at least three independent experiments are presented as the mean and standard deviation. The data were analyzed using Student's *t* test. *P*<0.05 compared with the control condition was considered significant.

## Results

### Effects of DMF/BSO Combined with Irradiation on Endogenous GSH Levels of SQ20B Cells

In the first set of experiments (Table 1A), we have quantified the total GSH content in SQ20B cells treated under different conditions. When used in combination, DMF and BSO treatment resulted in total GSH depletion after 4 h of incubation with a slow restoration of the GSH level at 24 h. A 4 h DMF/BSO pretreatment combined with X-ray or carbon ion exposure enabled the stabilization of the GSH depletion and the inhibition of glutathione resynthesis induced after irradiation (Table 1B). No significant variations of oxidized glutathione were detected under our experimental conditions (data not shown). This protocol was therefore considered as optimal to efficiently and transiently deplete SQ20B GSH stores.

**Table 1:** The endogenous glutathione content determined by HPLC analysis in SQ20B cells.

A.			
Total Glutathione (nmol/mg proteins) in SQ20B cells			
DMF/BSO	−	+	+
Time post-treatment	0 h	4 h	24 h
Glutathione content	67,47±1,47	4,48±0,59	18,96±0,45

B.				
Total Glutathione (nmol/mg proteins) in SQ20B cells				
DMF/BSO	−	−	+	+
Time post-treatment	4 h	24 h	4 h	24 h
X-rays	64,21±3,38	45,86±5,17	2,4±0,35	24,11±2,89
Carbon ions	83,7±1,99	23,7±0,5	<detection theshold	23,2±0,08

Endogenous glutathione content was determined in SQ20B cells after 4 h of treatment with DMF (100 µM) and BSO (100 µM) (A). Panel B shows the endogenous glutathione level with and without 10 Gy of X-ray and 5 Gy of carbon ion irradiation with or without treatment with DMF/BSO. Results are expressed as mean ± S.D. for three different experiments in triplicate.

### Clonogenic Cell Survival

**Figure 1:**
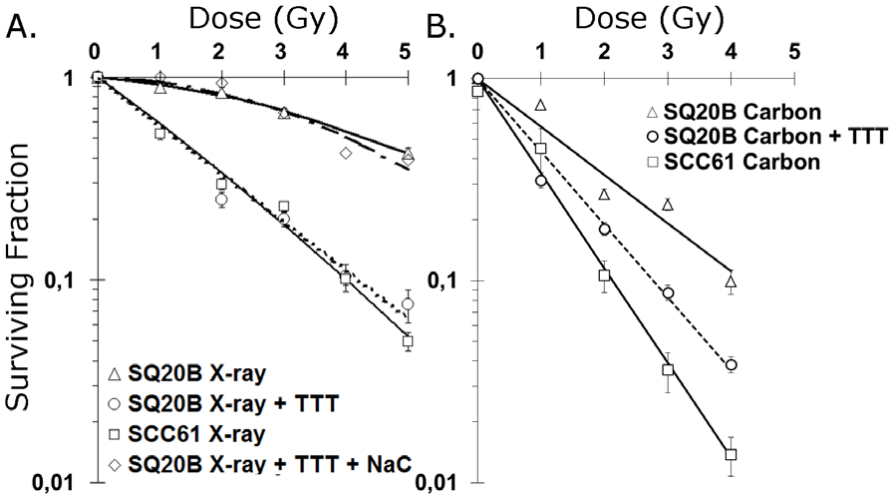
Clonogenic cell survival assay. SQ20B, DMF/BSO (TTT)-treated SQ20B, and SCC61 cells were exposed to X-ray radiation (A) or carbon ion radiation (B). The reverse effect of NaC over the glutathione depletion treatment was evaluated by coincubating DMF/BSO (TTT)-treated SQ20B with 5 mM of *N*-acetyl cysteine (NaC) (SQ20B+TTT+NaC) before X-ray radiation exposure.

The results for clonogenic cell survival in the radiosensitive SCC61 and the radioresistant SQ20B cell lines after X-ray or carbon ion irradiation are shown in Fig. 1. The exposure to carbon ions resulted in a lower surviving fraction compared with X-rays in both cell lines. The survival fraction at 2 Gy (SF2) was 0.81 (X-rays) and 0.33 (carbon ions) for SQ20B cells and 0.34 (X-rays) and 0.12 (carbon ions) for SCC61 cells. SQ20B cells were systematically more resistant than SCC61 cells, even in response to carbon ions. The relative biological effectiveness at the 10% survival level was 2.1 for SQ20B cells and 1.9 for SCC61 cells. In the presence of DMF/BSO, radiosensitization of resistant SQ20B cells occurred and the resultant SF2 (0.28 for X-rays and 0.19 for carbon ions) was found to be similar to that measured in the untreated sensitive SCC61 cell line. Such a radiosensitization was reversed in the presence of 5 µM NaC, a highly powerful antioxidant agent, as evidenced by the SF2 value (0.84) of treated SQ20B cells after X-ray irradiation.

### DNA DSB Analysis by γH2AX Assay

**Figure 2:**
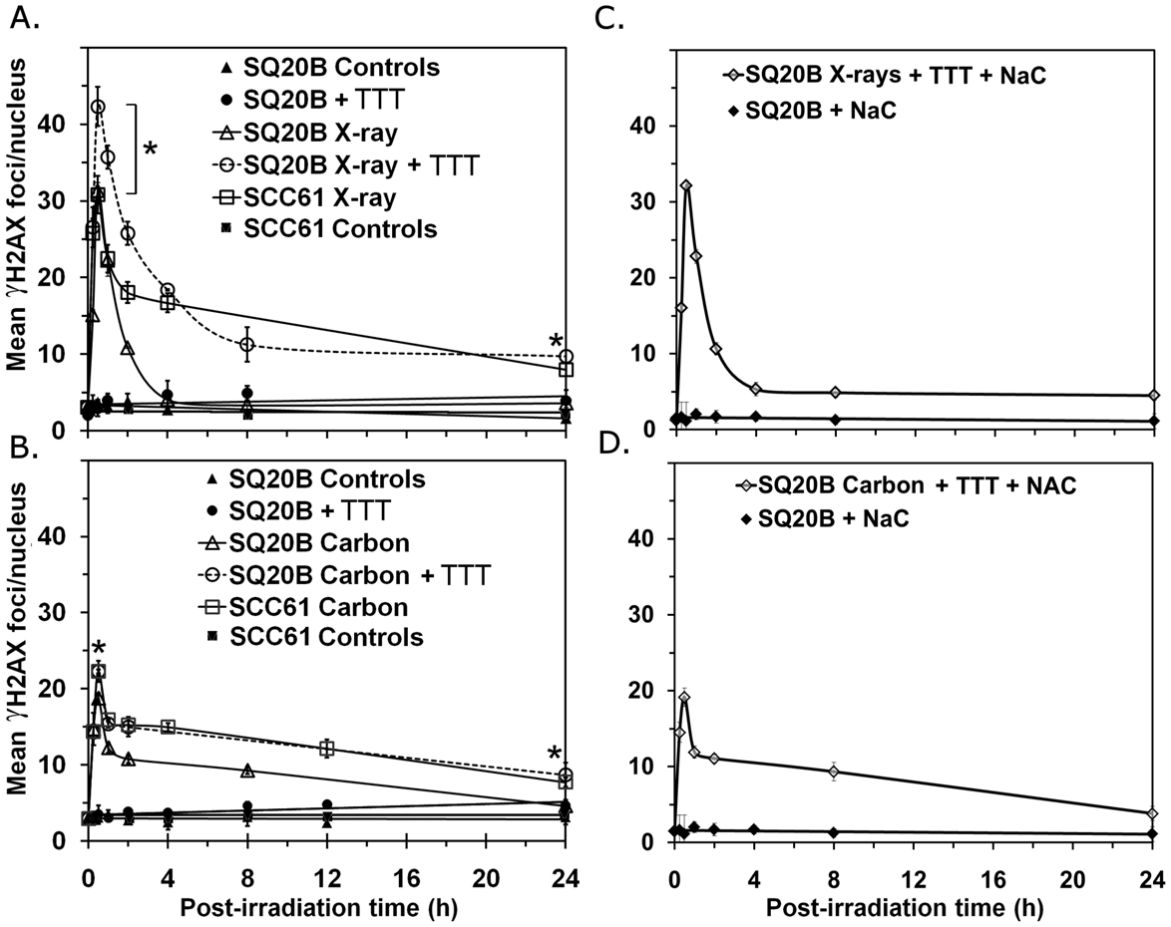
Kinetics of γH2AX foci. Cells were irradiated with 2 Gy of X-rays (A, C) or 1 Gy of carbon ions (B, D). ▴ SQ20B cells, ▵ SQ20B+irradiation, • SQ20B+GSH depletion, • SQ20B+GSH depletion+irradiation, □ SCC61+irradiation. In panels C and D, the reverse effect of *N*-acetyl cysteine in the presence of DMF/BSO treatment was assayed. One hundred cells were scored for each time and the measurements were made in triplicate and repeated three times. *P<0.05.

We first evaluated the efficiency of DSB repair according to the kinetics of γH2AX foci. As shown in Fig. 2*A*, the initial peak of γH2AX foci per cell after X-ray irradiation was similar in both the radioresistant SQ20B (31.3±1.0) cell line which displays the highest level of endogenous GSH and the radiosensitive SCC61 (30.8±2.4) cell line, thus suggesting that GSH content did not impact on the yield of DSB after X-ray irradiation. However, the kinetics of repair differed: repair was slower in the sensitive SCC61 cells and resulted in an accumulation of residual DSB (8 foci/nucleus) 24 h after irradiation. After carbon ion exposure (Fig. 2*B*), the maximum number of γH2AX foci per cell was lower than after X-ray irradiation (19±0.7 and 22.2±0.3 for SQ20B and SCC61 cell lines, respectively). In contrast to the X-ray irradiation response, the repair kinetics was similar between the two cell lines. Finally, the number of residual γH2AX foci measured in sensitive cells 24 h after carbon ion exposure was equivalent to that observed after the biological isodose of X-rays (7.7±0.6 foci/nucleus), whereas no residual DSB were measured in resistant cells after either type of irradiation.

Depleting the intracellular GSH level in SQ20B cells by DMF/BSO treatment did not cause any significant variation in the spontaneous DSB amount compared with control cells (0 to 5 foci) during the time course studied, whereas the combination with X-ray or carbon ion irradiation significantly affected the cellular response. The number of γH2AX foci increased significantly (*P*<0.05) 30 min after X-ray irradiation (42.3±2.3 foci versus 31.3±1.0 in irradiated SQ20B cells) and the repair kinetics were then slower than those observed in irradiated, but untreated SQ20B cells. The increase in the number of initial DNA lesions led to residual DSB (9.7±1.5 foci) at 24 h, a level similar to that measured in sensitive SCC61 cells. Interestingly, after carbon ion exposure, the response of GSH-depleted SQ20B cells matched perfectly that of irradiated SCC61 cells in terms of the initial DNA damage, repair kinetics, and residual DSB. These results indicate that the depletion of the endogenous GSH pool in resistant cells combined with X-ray or carbon ion exposure at a biologically equivalent dose led to the persistence of DNA lesions at a level similar to that in the sensitive SCC61 cells.

To confirm the role of redox changes on DNA damage, we incubated SQ20B cells with NaC. Under these experimental conditions, NaC did not induce γH2AX foci in control experiments (Fig. 2 C and D). In the presence of DMF/BSO treatment and NaC added 4 h before irradiation, the γH2AX measurements showed that the level of DSB and kinetics of repair match with irradiated, but untreated, SQ20B cells, for either type of irradiation. NaC may thus reverse the effect of GSH depletion.

### Single Strand Breaks and Oxidative DNA Lesions Measured by the SCGE Assay

**Figure 3:**
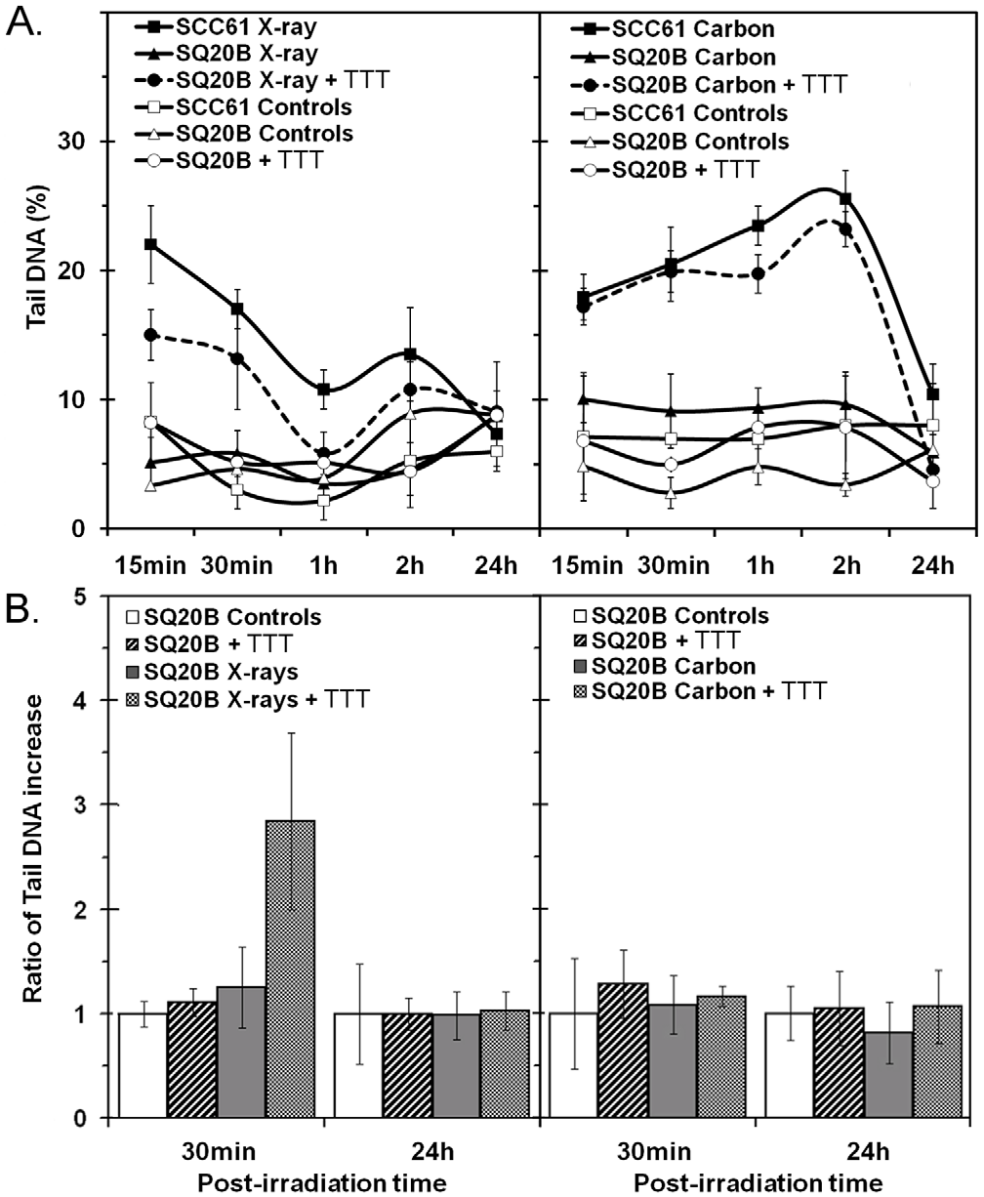
Comet assay. Mean percentage of DNA damage in SCC61, SQ20B, and DMF/BSO (TTT)-treated SQ20B cell lines after 10 Gy of X-ray or 5 Gy of carbon ion exposure. Comet assays were performed in alkaline conditions without (A) or in the presence of Fpg enzyme (B). ▵ SQ20B cells, ▴SQ20B+irradiation, • SQ20B+GSH depletion, ○ SQ20B+GSH depletion+irradiation, ▪ SCC61, □ SCC61+irradiation. *P<0.05.

The level of alkali-labile sites and SSB in DNA was investigated using the SCGE assay. As shown in Fig. 3*A*, sensitive SCC61 and resistant SQ20B cell lines clearly displayed distinct responses in terms of radioinduced SSB. No signal was obtained from SQ20B cells during the time studied with either type of irradiation. By contrast, SCC61 cells were highly responsive and showed a high level of breaks at the shortest times after irradiation. Rapid repair occurred after X-ray exposure, as shown by the decreasing number of breaks with time. Although carbon ion irradiation induced a similar initial percentage of tail DNA compared with X-ray irradiation, the repair kinetics in SCC61 cells was considerably slower and showed a sustained increase up to 2 h followed by a decrease for a longer time. Interestingly, GSH-depleted SQ20B cells showed a similar pattern to that observed for SCC61 cells and the rate was similar after exposure to either type of radiation. This suggests that high endogenous GSH levels protect DNA against radiation in SQ20B cells. In a second set of experiments, the percentage of tail DNA identified using SCGE alone was subtracted from that obtained after treatment with the Fpg enzyme to study the spatial distribution of oxidized bases. The results shown in Fig. 3*B* indicate that in SQ20B cells, X-ray or carbon ion irradiation did not modify the oxidation of DNA bases compared with controls. However, GSH-depleted SQ20B cells displayed more scattered oxidative damage at the shortest time after X-ray irradiation, whereas a less variable pattern of damage after exposure to carbon ions suggested the local production of free radicals.

### Radioinduced G2/M Phase Arrest and Accumulation of Cells in the Sub-G1 Phase after Cell Cycle Analysis

**Figure 4:**
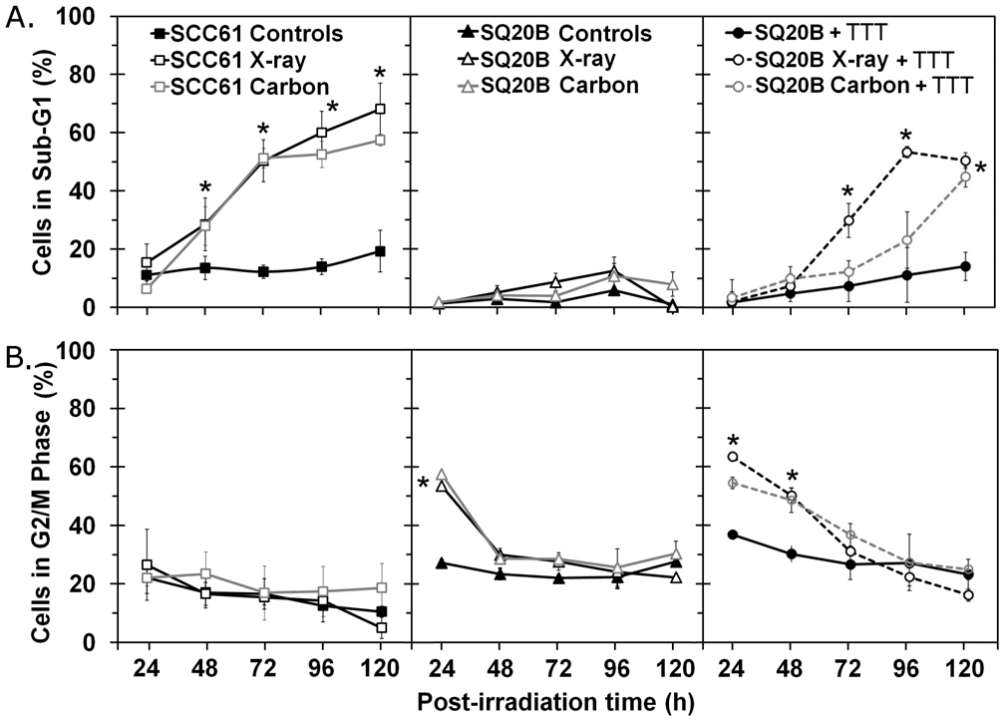
Cell cycle distribution. SCC61, SQ20B, and DMF/BSO (TTT)-treated SQ20B cells were exposed to X-rays or carbon ion radiation. (A) The percentage of cells in sub-G1 phase. (B) The percentage of cells in G2/M phase. ▴ SQ20B cells, ▵ SQ20B+5 Gy carbon ion radiation, ▵ SQ20B+10 Gy X-rays, • SQ20B+GSH depletion, ○ SQ20B+GSH depletion+5 Gy carbon ion radiation, ○ SQ20B+GSH-depletion+10 Gy X-rays, ▪ SCC61, □ SCC61+5 Gy carbon ion radiation, □ SCC61+10 Gy X-rays. *P<0.05.

To determine to what extent GSH depletion and the residual DSB could affect the cellular response to X-ray or carbon ion irradiation through cell cycle redistribution, the relative number of SCC61, SQ20B, and GSH-depleted SQ20B cells in the sub-G1 (Fig. 4*A*) and G2/M (Fig. 4*B*) phases was analyzed by flow cytometry. The sensitive SCC61 cells rapidly underwent apoptosis (approximately 32% of sub-G1 cells at 48 h, increasing to 68% at 120 h). By contrast, no significant level of apoptosis was measured in SQ20B cells after either type of irradiation. Instead, up to 55% of SQ20B cells were arrested at the G2/M checkpoint after 24 h following both types of irradiation, and these cells reentered the cell cycle after 48 h. GSH depletion of SQ20B cells increased the G2/M phase arrest, after which the cells were released and returned to the basal level only 72 h after irradiation. The relapse of G2/M arrest correlated with the increase in the percentage of apoptotic cells after X-ray irradiation (55% at maximum). This increase was slightly delayed (96 h) after carbon exposure but reached the same level at 120 h. These data indicate that the depletion of the endogenous pool of GSH influences the proportion of cells arrested at the G2/M checkpoint in culture and its duration in relation to radiation quality.

### Micronuclei Measurements Detected using the Cytome Assay

**Figure 5:**
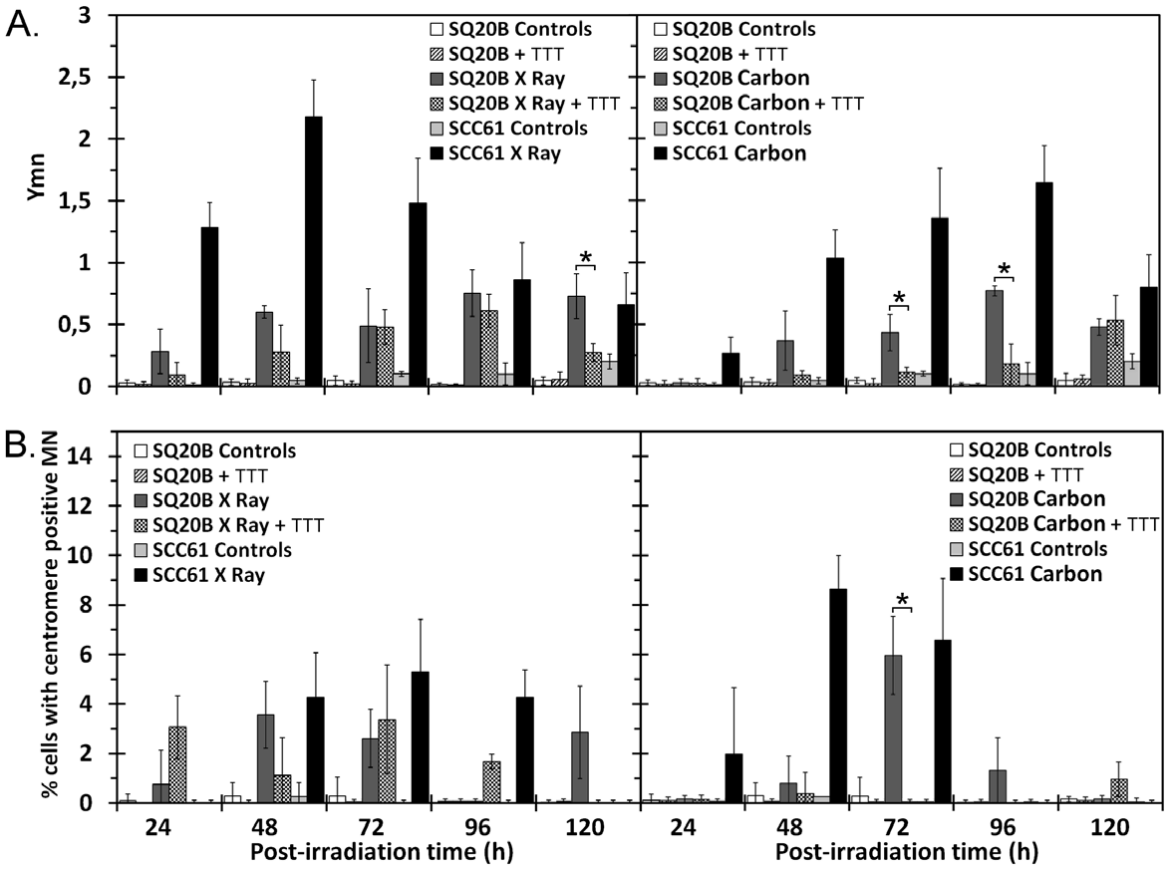
Micronucleus cytome assay. Yield of micronuclei (A) and centromere-positive micronuclei (B) in SCC61, SQ20B, and DMF/BSO (TTT)-treated SQ20B cell lines after 10 Gy of X-ray or 5 Gy of carbon ion irradiation. *P<0.05.

Unrepaired or misrepaired DNA damage can lead to chromosome changes in surviving cancer cells. The formation of MN, which contain a fragment of a chromosome/chromatid, may be linked to unrepaired DSB, thus reflecting a defect in repair. The yield of MN was estimated by calculating the Ymn value. As shown in Fig. 5*A*, the Ymn values after exposure to X-ray and carbon ion irradiation are correlated with the cell radiosensitivity. More MN were produced in sensitive SCC61 compared with SQ20B cells after both types of irradiation. The maximum yield in SCC61 cells did not differ significantly between the two types of irradiation (2.1±0.3 after X-ray irradiation and 1.68±0.3 after carbon ion irradiation). The maximum value was slightly delayed after carbon ion irradiation (96 h), a time corresponding to the triggering of apoptosis, as described above. By contrast, the yield of MN induced in resistant SQ20B cells did not exceed 0.75 and was similar for both types of irradiation. Although the radiosensitization of SQ20B cells through GSH depletion led to residual DSB identical to those observed in SCC61 cells after irradiation, it did not induce the same pattern of MN. The Ymn values measured in GSH-depleted SQ20B were equal to those in undepleted SQ20B cells after X-irradiation (excepted at 120 h post-irradiation), but were lower after carbon ion exposure for the majority of the kinetic time points. Finally, only carbon ion irradiation induced an obvious decrease in the number of radioinduced MN in GSH-depleted SQ20B cells.

In a second set of experiments, chromosome/chromatid loss (identified as the centromere-positive MN, c+MN) was estimated (Fig. 5*B*). The percentage of cells with c+MN was low after X-ray irradiation and did not differ between sensitive and resistant cells (maximum ∼4% to 5%). After carbon irradiation, the c+MN level was low except at the time corresponding to the induction of apoptosis in SCC61 and to the G2/M phase arrest relapse in SQ20B cells, whereas a marked increase was measured between 48 and 72 h. At these times, the number of c+MN was twice as high after carbon irradiation compared with X-ray exposure (approximately 8% of cells). This might correspond to a specific signature of carbon ion irradiation. Finally, no more c+MN were observed in GSH-depleted SQ20B cells after carbon ion exposure.

### Chromosome Rearrangements Measured using the Cytome Assay

**Figure 6:**
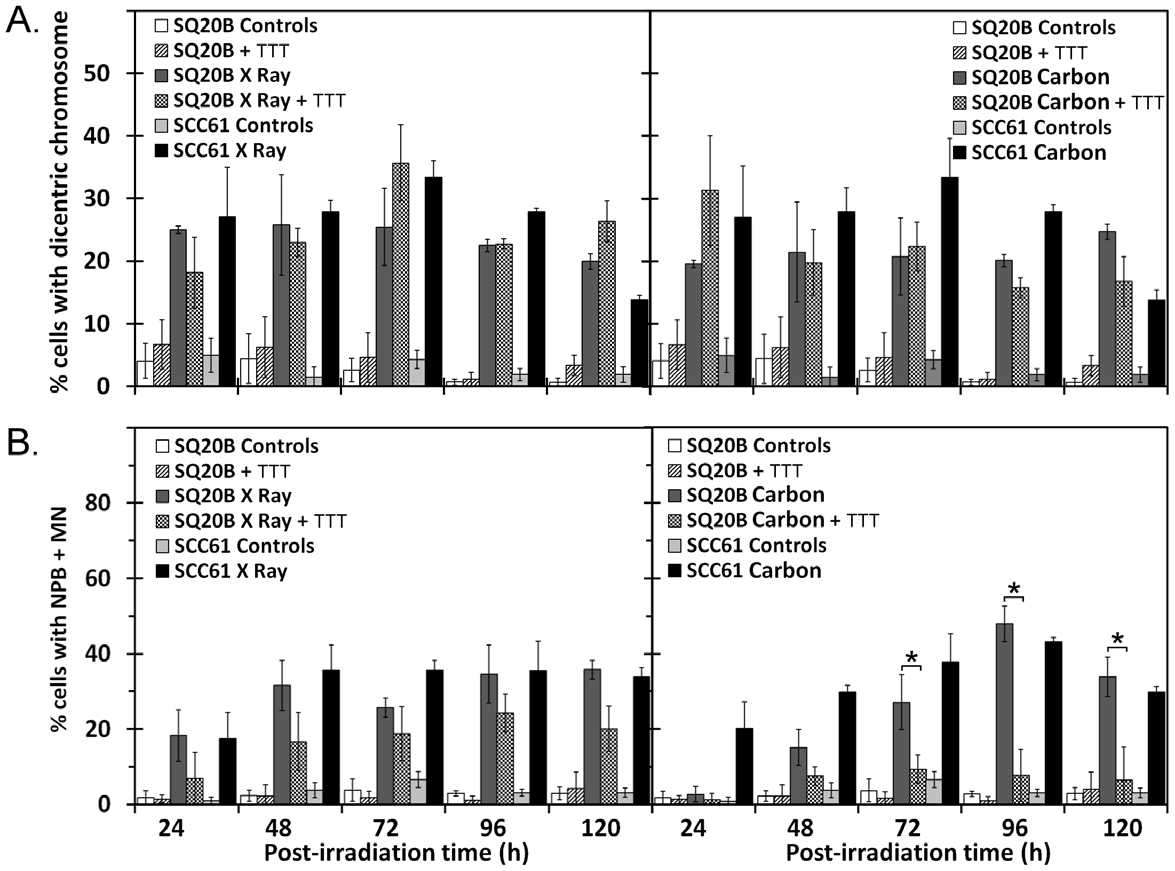
Chromosomal rearrangements. Percentage of cells displaying chromosomal rearrangements including dicentric chromosome formation (A) and complex rearrangements (B). The effects were investigated in SCC61, SQ20B, and DMF/BSO (TTT)-treated SQ20B cell lines after 10 Gy of X-ray or 5 Gy of carbon ion irradiation. *P<0.05.

Two types of rearrangements were considered: apparently dicentric chromosomes, which were visualized as nucleoplasmic bridges (NPB), and the more complex rearrangements, which were visualized by simultaneous appearance of NPB and MN. As shown in Fig. 6*A*, the frequency of dicentric chromosomes was similar in SCC61 and SQ20B cell lines (25–30% of cells) after X-ray or carbon ion exposure. GSH depletion in SQ20B cells did not alter significantly any values regardless of the type of irradiation. Interestingly, these findings were independent of the cell cycle distribution or G2/M arrest. The expression of dicentric chromosomes in surviving cancer cells seemed to be independent of the intrinsic radiosensitivity and type of radiation. No differences were observed in complex rearrangements between SCC61 and SQ20B, as evidenced by the simultaneous observation of NPB and MN after X-ray exposure (Fig. 6*B*) in about 35% of cells throughout the time studied. GSH depletion in SQ20B cells had no significant effect on this type of rearrangement. By contrast, high-LET radiation led to a progressive increase in the percentage of NPB+MN-positive cells, which peaked 96 h after irradiation at about 45% of positive SCC61 and SQ20B cells. GSH depletion in SQ20B cells led to a strong and significant decrease in CCs at all times. The induction of complex rearrangements was independent of cell radiosensitivity, but these rearrangements differed according to the radiation type and GSH depletion in surviving cancer cells.

## Discussion

The aim of our study was to highlight the relationship between the nature of DNA damage and the consecutive chromosomal aberrations in response to low- and high-LET irradiation after a transient depletion of endogenous glutathione in resistant HNSCC cancer cells. To address this issue, X-ray and carbon ion irradiation were performed at a biologically equivalent dose to focus on events leading to an equivalent level of cell death (evaluated with the relative biological effectiveness and clonogenic assays); this was performed to enable a comparison of the nature of DNA damage and the consequences on the transmission of chromosomal changes according to the type of radiation, radioresistance status, and endogenous GSH content.

The resistant and sensitive HNSCC cell lines displayed different responses in terms of DNA lesions and repair capacity in relation to their GSH content. In response to X-ray exposure, the resistant SQ20B cell line, with a higher endogenous GSH content, showed a higher DNA repair capacity that enabled fast disappearance of DSB and SSB. By contrast, the repair capacity of sensitive SCC61 cells was slower and led to the persistence of residual DSB. These results confirm the previously suggested notion [18], [22], [23] that GSH level correlates with DNA repair capacity. By contrast, a biologically equivalent dose of carbon ions (1 Gy carbon ions compared with 2 Gy X-rays) induced fewer initial breaks. The repair kinetics were slower than after X-ray exposure, as confirmed by Schmid et al. [40], and were equal in both cell lines. Lesions were more difficult to repair, and the endogenous GSH level had no influence on the repair capacity after high-LET radiation, as previously reported [23]. These observations support the concept that breaks produced by particle tracks are more clustered and complex than are those produced by X-rays [2], [41]. Interestingly, regardless of the number and the complexity of the initial lesions, sensitive cells were characterized by the same level of residual DSB 24 h after the equivalent biological dose of X-rays and carbon ions. This damage reflects defects in the repair processes and correlates with apoptotic induction [42].

The transient GSH depletion in SQ20B cells led to the persistence of equal numbers of residual DSB after low- or high-LET exposure performed at a biologically equivalent dose. Interestingly, the number of unrepaired DSB and the percentage of apoptotic cells were similar to those measured in radiosensitive cells. The sensitizing effect induced by GSH depletion confirms its role in the mechanisms of radioresistance [11], [43] against both high- and low-LET radiation. To focus on the DNA radioprotective role of GSH, shorter kinetics points should be compared. After X-ray irradiation, GSH depletion increased the number of sparse DSB and SSB, and oxidized DNA bases. Experiments using NaC demonstrated the reverse effect of the GSH depletion on DSB and confirmed that redox changes induced by this depletion are responsible for the effect on DNA damage. The appearance of these sparse lesions corroborates the predominance of an indirect effect that produces long-life free radicals that react with DNA to generate DSB [22], [44]. By contrast, after carbon ion irradiation, DNA lesions such as DSB or SSB require a much longer time to repair. The reverse effect of NaC suggests the involvement of oxidative stress in the induction of DNA lesions. Additional experiments are needed to demonstrate the involvement of oxidative stress. However, our results suggest that GSH modulation impacts more on the quality of DNA lesions than on the quantity. The distribution of oxidized bases was not scattered, but appeared to colocalize with SSB, or even DSB, as evidenced by the results obtained after the addition of Fpg enzyme during the comet assays. The hypothesis of localized clusters of oxidized bases is strengthened by the work of Bergeron et al. [45], who showed that such lesions are refractory to excision-repair systems. This suggests that short-life free radicals are produced along the carbon ion track and that these free radicals increase the complexity of DNA lesions after GSH depletion combined with carbon ion irradiation. These data confirm the importance of an indirect effect at a local scale in response to carbon ion irradiation; in contrast to previous reports [19], [22], our data demonstrate the protective role of GSH against oxidative clustered DNA damage formation after carbon ion radiation.

Most studies have investigated the radioprotective role of GSH by adding exogenous GSH or other scavengers, but without considering the endogenous level of GSH in their cellular model [18], [19], [22], [46]. Considering the results presented in this paper, the inclusion of this parameter now seems to be fundamental. In our cellular models, we agreed that a high endogenous GSH content correlates with a faster repair process, suggesting that GSH helps in DSB rejoining, as reported previously by others [18], [19], [22], [23] who did not propose any speculation about a possible specific intrinsic mechanism. Our data did not enable to further speculate about rejoining. However, GSH depletion modifies the kinetics of repair after X-ray radiation, suggesting that GSH had a direct effect on the repair systems in our cellular models and probably on DNA damage recognition. The relationship between DNA repair capacity and endogenous GSH level probably proceeds from inducing cell adaptation to protecting against oxidative stress [12]. In SQ20B cells, transient GSH depletion favored radiosensitization through the induction of supernumerary and highly complex lesions after X-ray and carbon ion irradiation, respectively, leading to a defect in repair, as observed in sensitive SCC61 cells.

DNA damage and its management are crucial in determining cell fate. The more severe lesions should guide cells toward death, and misrepaired lesions should lead to chromosomal changes. Cancer cells have a highly rearranged genome and may survive even after a part of the genome is lost or modified. By contrast, transmission of chromosomal changes to cancer cell progeny may lead to adaptation and acquisition of additional resistance mechanisms. The analyses of cell cycle distribution after both types of radiation showed that resistant cells displayed G2/M arrest before release and survival, whereas sensitive SCC61 cells died rapidly. GSH depletion combined with irradiation induces a more longer lasting G2/M arrest, during which the repair of persistent DSB is attempted [24], [47]. The direct consequence is that first mitosis occurs with delays. Using the cytome assay, we have assessed the consequences of misrepaired and residual DNA lesions from the first mitosis after irradiation for up to five days. As suggested by Johannes et al. [48], the cytome assay gives a general overview of possible transmissible chromosomal changes in cells that survive after mitosis.

Four classes of CCs were considered. First, in contrast to a previous study [4], our analysis showed that dicentric chromosome expression (NPB formation) was unchanged regardless of the intrinsic radiosensitivity of the cell line and the type of radiation applied. We also demonstrated that dicentric chromosome expression did not vary according to the endogenous GSH level. Second, complex rearrangements (simultaneous expression of NPB and MN) were considered. Contrary to a previous suggestion [4], such complex aberrations do not depend on the sensitivity of cells to radiation, but do depend on the type of radiation and the radioinduced DNA lesions produced. We demonstrated in GSH-depleted SQ20B cells that an increase in the number of sparse DNA lesions after X-ray irradiation did not modify the expression of complex rearrangements. By contrast, after GSH depletion combined with carbon ion exposure in SQ20B cells, the increasing complexity of lesions decreased the number of complex rearrangements at the same time as triggering apoptosis, but it was not equivalent to what happened in SCC61 cells. These findings suggest that only a modification of the complexity along with highly clustered lesions could prevent the transmission of complex rearrangements to surviving cancer cells. This may be one advantage of hadrontherapy, i.e. a treatment minimizing genomic instability and tumor escape.

Among the four classes of CCs observed in this study, chromatid/chromosome breakage or loss should be considered a critical parameter. In this set of experiments, high-LET radiation induced a higher frequency of MN with centromeres in SQ20B and SCC61 cell lines compared with X-ray radiation. To our knowledge, such a molecular signature has, up to now, never been reported [2], [19]. Therefore, we propose that chromatid/chromosome loss corresponds to a specific “fingerprint” of exposure to carbon ions [3]. We note that cancer cells display different numbers of chromosomes that may influence the yield of MN, as higher chromosome numbers may be linked to higher MN production. However, as a clear estimation of chromosome number was not possible at the time of experimentation, it is difficult to weight these values. Globally, SQ20B cells are known to display a higher chromosome number than SCC61.cells [49]. The sensitive SCC61 cell line displayed a higher level of MN compared with the resistant SQ20B cell line. The percentage of cells with MN reflected the radiosensitivity of the cell lines assayed.

Among the possible explanations, it is likely that the G2/M arrest mechanism allows resistant cells to repair lesions [50], whereas misrepaired or unrepaired DSB lead to MN in sensitive cells. A decrease in the frequency of deletions was previously associated with high levels of GSH [18], [19], [23]. Our results in SQ20B cells, which displayed a high GSH content, confirm these previous findings. Pretreatment of SQ20B cells with DMF/BSO did not affect the MN ratio after X-ray irradiation compared with the response in untreated cells, whereas exposure to carbon ion radiation markedly decreased the number of MN. In all cases, the GSH-depleted SQ20B cells did not show the same profile of MN as the SCC61 cells, suggesting that the MN profile is dependent on the intrinsic radioresistance of the SQ20B cell line. This finding may contradict the data from previous studies based on sister chromatid staining that reported an increase in deletion frequency after GSH depletion [23] or a decrease after GSH addition [18] in human lymphocytes. This discrepancy may reflect the status of the cells, which were studied before the completion of the first metaphase. The G2/M arrest observed in GSH-depleted SQ20B cells, but not in SCC61 cells, may thus explain the differences in the MN profiles. During G2/M arrest, cancer cells try to repair DNA, correctly or incorrectly, and then undergo mitosis or apoptosis [51]. We hypothesize that GSH modulation is part of regulation of the balance between cell cycle arrest and damage repair on the one hand and initiation of cell death on the other. GSH depletion may reactivate the correct function of DNA-damage checkpoints, favoring cell death before mitosis, and may minimize the transmission of MN in the progeny following carbon ion irradiation, but not after X-ray irradiation. The prevention of transmissible MN and rearrangements is essential for guaranteeing the absence of chromosomal changes and consequently for limiting the genomic instability in surviving cells.

## Conclusion

In this work, we have demonstrated for the first time that GSH modulation potentiates the effect of radiation on DNA by inducing a greater number of sparse lesions after X-ray irradiation and higher complex damage after carbon ion irradiation in resistant HNSCC cancer cells. Both types of radiation induced rearrangements in the surviving cells, and chromosome/chromatid loss appeared as a specific signature of carbon ion exposure in sensitive and resistant cells. In resistant cancer cells, experiments using GSH depletion combined with carbon ion irradiation showed that only an increase in DNA lesion complexity could be a key control to limit the transmission of chromosomal changes after the first mitosis such as MN or complex rearrangements in surviving cells. This is not observed in sensitive cells, the process being certainly related to different mechanisms of cell cycle arrest. Taken together, our results suggest that a combination of hadrontherapy with GSH depletion should significantly improve patient outcomes by minimizing genomic instability and improving the local tumor control.

## References

[1] Fokas E, Kraft G, An H, Engenhart-Cabillic R (2009) Ion beam radiobiology and cancer: Time to update ourselves. Biochimica et Biophysica Acta (BBA) - Reviews on Cancer 1796: 216–229 doi:10.1016/j.bbcan.2009.07.005. 19682551 10.1016/j.bbcan.2009.07.005

[2] Hamada N (2009) Recent insights into the biological action of heavy-ion radiation. J Radiat Res 50: 1–9. 18838844 10.1269/jrr.08070

[3] Ritter S, Durante M (2010) Heavy-ion induced chromosomal aberrations: A review. Mutat Res 701: 38–46 doi:10.1016/j.mrgentox.2010.04.007. 20398789 10.1016/j.mrgentox.2010.04.007

[4] Virsik-Köpp P, Hofman-Huether H (2004) Chromosome aberrations induced by high-LET carbon ions in radiosensitive and radioresistant tumour cells. Cytogenet Genome Res 104: 221–226 doi:10.1159/000077493. 15162042 10.1159/000077493

[5] Lee R, Sommer S, Hartel C, Nasonova E, Durante M, et al (2010) Complex exchanges are responsible for the increased effectiveness of C-ions compared to X-rays at the first post-irradiation mitosis. Mutat Res 701: 52–59 doi:10.1016/j.mrgentox.2010.03.004. 20298802 10.1016/j.mrgentox.2010.03.004

[6] Anderson GR (2001) Genomic instability in cancer. Current Science 81.

[7] Weichselbaum RR, Dahlberg W, Beckett M, Karrison T, Miller D, et al (1986) Radiation-resistant and repair-proficient human tumor cells may be associated with radiotherapy failure in head- and neck-cancer patients. Proceedings of the National Academy of Sciences 83: 2684–2688. 10.1073/pnas.83.8.2684 PMC323364 3458227

[8] Wiseman SM, Stoler DL, Anderson GR (2004) The role of genomic instability in the pathogenesis of squamous cell carcinoma of the head and neck. Surg Oncol Clin N Am 13: 1–11 doi:10.1016/S1055-3207(03)00118-2. 15062358 10.1016/S1055-3207(03)00118-2

[9] Mizoe J-E, Tsujii H, Kamada T, Matsuoka Y, Tsuji H, et al (2004) Dose escalation study of carbon ion radiotherapy for locally advanced head-and-neck cancer. Int J Radiat Oncol Biol Phys 60: 358–364 doi:10.1016/j.ijrobp.2004.02.067. 15380567 10.1016/j.ijrobp.2004.02.067

[10] Jereczek-Fossa BA, Krengli M, Orecchia R (2006) Particle beam radiotherapy for head and neck tumors: radiobiological basis and clinical experience. Head Neck 28: 750–760 doi:10.1002/hed.20448. 16804876 10.1002/hed.20448

[11] Estrela JM, Ortega A, Obrador E (2006) Glutathione in cancer biology and therapy. Crit Rev Clin Lab Sci 43: 143–181 doi:10.1080/10408360500523878. 16517421 10.1080/10408360500523878

[12] Trachootham D, Alexandre J, Huang P (2009) Targeting cancer cells by ROS-mediated mechanisms: a radical therapeutic approach? Nat Rev Drug Discov 8: 579–591 doi:10.1038/nrd2803. 19478820 10.1038/nrd2803

[13] Diehn M, Cho RW, Lobo NA, Kalisky T, Dorie MJ, et al (2009) Association of reactive oxygen species levels and radioresistance in cancer stem cells. Nature 458: 780–783 doi:10.1038/nature07733. 19194462 10.1038/nature07733 PMC2778612

[14] Boivin A, Hanot M, Malesys C, Maalouf M, Rousson R, et al (2011) Transient Alteration of Cellular Redox Buffering before Irradiation Triggers Apoptosis in Head and Neck Carcinoma Stem and Non-Stem Cells. PLoS ONE 6: e14558 doi:10.1371/journal.pone.0014558. 21283807 10.1371/journal.pone.0014558 PMC3023721

[15] Zhao Y, Seefeldt T, Chen W, Carlson L, Stoebner A, et al (2009) Increase in thiol oxidative stress via glutathione reductase inhibition as a novel approach to enhance cancer sensitivity to X-ray irradiation. Free Radic Biol Med 47: 176–183 doi:10.1016/j.freeradbiomed.2009.04.022. 19397999 10.1016/j.freeradbiomed.2009.04.022 PMC2745482

[16] Cadet J, Ravanat J-L, Taverna-Porro M, Menoni H, Angelov D (2012) Oxidatively generated complex DNA damage: tandem and clustered lesions. Cancer letters 10.1016/j.canlet.2012.04.005 22542631

[17] Mansour HH, Hafez HF, Fahmy NM, Hanafi N (2008) Protective effect of N-acetylcysteine against radiation induced DNA damage and hepatic toxicity in rats. Biochem Pharmacol 75: 773–780 doi:10.1016/j.bcp.2007.09.018. 18028880 10.1016/j.bcp.2007.09.018

[18] Pujari G, Berni A, Palitti F, Chatterjee A (2009) Influence of glutathione levels on radiation-induced chromosomal DNA damage and repair in human peripheral lymphocytes. Mutat Res 675: 23–28 doi:10.1016/j.mrgentox.2009.02.001. 19386243 10.1016/j.mrgentox.2009.02.001

[19] Pujari G, Sarma A, Chatterjee A (2010) The influence of reduced glutathione on chromosome damage induced by X-rays or heavy ion beams of different LETs and on the interaction of DNA lesions induced by radiations and bleomycin. Mutat Res 696: 154–159 doi:10.1016/j.mrgentox.2010.01.006. 20100593 10.1016/j.mrgentox.2010.01.006

[20] Tulard A, Hoffschir F, de Boisferon FH, Luccioni C, Bravard A (2003) Persistent oxidative stress after ionizing radiation is involved in inherited radiosensitivity. Free Radical Biology and Medicine 35: 68–77 doi:10.1016/S0891-5849(03)00243-0. 12826257 10.1016/s0891-5849(03)00243-0

[21] Heine T, Glatt H, Epe B (2006) Human cytochrome P450 reductase can act as a source of endogenous oxidative DNA damage and genetic instability. Free Radical Biology and Medicine 40: 801–807 doi:10.1016/j.freeradbiomed.2005.10.033. 16520232 10.1016/j.freeradbiomed.2005.10.033

[22] Fujii Y, Kato TA, Ueno A, Kubota N, Fujimori A, et al (2010) Ascorbic acid gives different protective effects in human cells exposed to X-rays and heavy ions. Mutat Res 699: 58–61 doi:10.1016/j.mrgentox.2010.04.003. 20394838 10.1016/j.mrgentox.2010.04.003

[23] Dutta A, Chakraborty A, Saha A, Ray S, Chatterjee A (2005) Interaction of radiation- and bleomycin-induced lesions and influence of glutathione level on the interaction. Mutagenesis 20: 329–335 doi:10.1093/mutage/gei046. 16014360 10.1093/mutage/gei046

[24] Ray S, Chatterjee A (2006) Influence of endogenous glutathione level on X-ray induced cell cycle delay in human lymphocytes. Cell Prolif 39: 37–47 doi:10.1111/j.1365-2184.2006.00365.x. 16426421 10.1111/j.1365-2184.2006.00365.x PMC6496169

[25] Suzuki M, Kase Y, Kanai T, Ando K (1998) Correlation between cell death and induction of non-rejoining PCC breaks by carbon-ion beams. Adv Space Res 22: 561–568. 11542786 10.1016/s0273-1177(98)00078-7

[26] Hamada N, Hara T, Funayama T, Sakashita T, Kobayashi Y (2008) Energetic heavy ions accelerate differentiation in the descendants of irradiated normal human diploid fibroblasts. Mutat Res 637: 190–196 doi:10.1016/j.mrfmmm.2007.07.002. 17716694 10.1016/j.mrfmmm.2007.07.002

[27] Durante M (2005) Biomarkers of space radiation risk. Radiat Res 164: 467–473. 16187751 10.1667/rr3359.1

[28] Fenech M (2006) Cytokinesis-block micronucleus assay evolves into a “cytome” assay of chromosomal instability, mitotic dysfunction and cell death. Mutat Res 600: 58–66 doi:10.1016/j.mrfmmm.2006.05.028. 16822529 10.1016/j.mrfmmm.2006.05.028

[29] Fenech M, Kirsch-Volders M, Natarajan AT, Surralles J, Crott JW, et al (2011) Molecular mechanisms of micronucleus, nucleoplasmic bridge and nuclear bud formation in mammalian and human cells. Mutagenesis 26: 125–132 doi:10.1093/mutage/geq052. 21164193 10.1093/mutage/geq052

[30] Heddle JA, Fenech M, Hayashi M, MacGregor JT (2011) Reflections on the development of micronucleus assays. Mutagenesis 26: 3–10 doi:10.1093/mutage/geq085. 20980366 10.1093/mutage/geq085

[31] Vral A, Fenech M, Thierens H (2011) The micronucleus assay as a biological dosimeter of in vivo ionising radiation exposure. Mutagenesis 26: 11–17 doi:10.1093/mutage/geq078. 21164177 10.1093/mutage/geq078

[32] Mateuca R, Lombaert N, Aka PV, Decordier I, Kirsch-Volders M (2006) Chromosomal changes: induction, detection methods and applicability in human biomonitoring. Biochimie 88: 1515–1531 doi:10.1016/j.biochi.2006.07.004. 16919864 10.1016/j.biochi.2006.07.004

[33] Fenech M (2009) A lifetime passion for micronucleus cytome assays–reflections from Down Under. Mutat Res 681: 111–117 doi:10.1016/j.mrrev.2008.11.003. 19100861 10.1016/j.mrrev.2008.11.003

[34] Maalouf M, Alphonse G, Colliaux A, Beuve M, Trajkovic-Bodennec S, et al (2009) Different Mechanisms of Cell Death in Radiosensitive and Radioresistant P53 Mutated Head and Neck Squamous Cell Carcinoma Cell Lines Exposed to Carbon Ions and X-Rays. Int J Radiat Oncol Biol Phys 74: 200–209 doi:10.1016/j.ijrobp.2009.01.012. 19362238 10.1016/j.ijrobp.2009.01.012

[35] Beuve M, Alphonse G, Maalouf M, Colliaux A, Battiston-Montagne P, et al (2008) Radiobiologic Parameters and Local Effect Model Predictions for Head-and-Neck Squamous Cell Carcinomas Exposed to High Linear Energy Transfer Ions. Int J Radiat Oncol Biol Phys 71: 635–642 doi:10.1016/j.ijrobp.2007.10.050. 18234427 10.1016/j.ijrobp.2007.10.050

[36] Alphonse G, Aloy MT, Broquet P, Gerard JP, Louisot P, et al (2002) Ceramide induces activation of the mitochondrial/caspases pathway in Jurkat and SCC61 cells sensitive to gamma-radiation but activation of this sequence is defective in radioresistant SQ20B cells. Int J Radiat Biol 78: 821–835 doi:10.1080/09553000210153943. 12428923 10.1080/09553000210153943

[37] Hanot M, Hoarau J, Carrière M, Angulo JF, Khodja H (2009) Membrane-Dependent Bystander Effect Contributes to Amplification of the Response to Alpha-Particle Irradiation in Targeted and Nontargeted Cells. International Journal of Radiation OncologyBiologyPhysics 75: 1247–1253 doi:10.1016/j.ijrobp.2009.07.014. 10.1016/j.ijrobp.2009.07.014 19857788

[38] Tice RR, Agurell E, Anderson D, Burlinson B, Hartmann A, et al (2000) Single cell gel/comet assay: guidelines for in vitro and in vivo genetic toxicology testing. Environ Mol Mutagen 35: 206–221. 10737956 10.1002/(sici)1098-2280(2000)35:3<206::aid-em8>3.0.co;2-j

[39] Mateuca R, Lombaert N, Aka PV, Decordier I, Kirsch-Volders M (2006) Chromosomal changes: induction, detection methods and applicability in human biomonitoring. Biochimie 88: 1515–1531 doi:10.1016/j.biochi.2006.07.004. 16919864 10.1016/j.biochi.2006.07.004

[40] Schmid TE, Dollinger G, Beisker W, Hable V, Greubel C, et al (2010) Differences in the kinetics of gamma-H2AX fluorescence decay after exposure to low and high LET radiation. Int J Radiat Biol 86: 682–691 doi:10.3109/09553001003734543. 20569192 10.3109/09553001003734543

[41] Shikazono N, Noguchi M, Fujii K, Urushibara A, Yokoya A (2009) The yield, processing, and biological consequences of clustered DNA damage induced by ionizing radiation. J Radiat Res 50: 27–36. 19218779 10.1269/jrr.08086

[42] Roos WP, Kaina B (2006) DNA damage-induced cell death by apoptosis. Trends Mol Med 12: 440–450 doi:10.1016/j.molmed.2006.07.007. 16899408 10.1016/j.molmed.2006.07.007

[43] Miura M, Sasaki T (1991) Role of glutathione in the intrinsic radioresistance of cell lines from a mouse squamous cell carcinoma. Radiat Res 126: 229–236. 2023994

[44] Koyama S, Kodama S, Suzuki K, Matsumoto T, Miyazaki T, et al (1998) Radiation-induced long-lived radicals which cause mutation and transformation. Mutat Res 421: 45–54. 9748497 10.1016/s0027-5107(98)00153-5

[45] Bergeron F, Auvré F, Radicella JP, Ravanat J-L (2010) HO* radicals induce an unexpected high proportion of tandem base lesions refractory to repair by DNA glycosylases. Proc Natl Acad Sci USA 107: 5528–5533 doi:10.1073/pnas.1000193107. 20212167 10.1073/pnas.1000193107 PMC2851781

[46] Ray S, Chatterjee A (2007) Influence of glutathione on the induction of chromosome aberrations, delay in cell cycle kinetics and cell cycle regulator proteins in irradiated mouse bone marrow cells. Int J Radiat Biol 83: 347–354 doi:10.1080/09553000701317887. 17457759 10.1080/09553000701317887

[47] Odom RY, Dansby MY, Rollins-Hairston AM, Jackson KM, Kirlin WG (2009) Phytochemical induction of cell cycle arrest by glutathione oxidation and reversal by N-acetylcysteine in human colon carcinomacarcinoma cells. Nutr Cancer 61: 332–339 doi:10.1080/01635580802549982. 19373606 10.1080/01635580802549982 PMC2749979

[48] Johannes C, Dixius A, Pust M, Hentschel R, Buraczewska I, et al (2010) The yield of radiation-induced micronuclei in early and late-arising binucleated cells depends on radiation quality. Mutat Res 701: 80–85 doi:10.1016/j.mrgentox.2010.05.005. 20472094 10.1016/j.mrgentox.2010.05.005

[49] Smeets MF, Mooren EH, Abdel-Wahab AH, Bartelink H, Begg AC (1994) Differential repair of radiation-induced DNA damage in cells of human squamous cell carcinoma and the effect of caffeine and cysteamine on induction and repair of DNA double-strand breaks. Radiat Res 140: 153–160. 7938462

[50] Ishikawa K, Ishii H, Saito T (2006) DNA damage-dependent cell cycle checkpoints and genomic stability. DNA Cell Biol 25: 406–411 doi:10.1089/dna.2006.25.406. 16848682 10.1089/dna.2006.25.406

[51] Stracker TH, Usui T, Petrini JHJ (2009) Taking the time to make important decisions: The checkpoint effector kinases Chk1 and Chk2 and the DNA damage response. DNA Repair 8: 1047–1054. 19473886 10.1016/j.dnarep.2009.04.012 PMC2725228

